# From the lab to the field: Self-stratifying microbial fuel cells stacks directly powering lights

**DOI:** 10.1016/j.apenergy.2020.115514

**Published:** 2020-11-01

**Authors:** Xavier Alexis Walter, Jiseon You, Jonathan Winfield, Ugnius Bajarunas, John Greenman, Ioannis A. Ieropoulos

**Affiliations:** Bristol BioEnergy Centre, Bristol Robotics Laboratory, T-Block, Frenchay Campus, University of the West of England (UWE), Bristol BS16 1QY, United Kingdom

**Keywords:** Self-stratifying microbial fuel cell, Urine treatment, Scaling-up, Power source, Practical applications

## Abstract

The microbial fuel cell (MFC) technology relies on energy storage and harvesting circuitry to deliver stable power outputs. This increases costs, and for wider deployment into society, these should be kept minimal. The present study reports how a MFC system was developed to continuously power public toilet lighting, with for the first time no energy storage nor harvesting circuitry. Two different stacks, one consisting of 15 and the other 18 membrane-less MFC modules, were operated for 6 days and fuelled by the urine of festival goers at the 2019 Glastonbury Music Festival. The 15-module stack was directly connected to 2 spotlights each comprising 6 LEDs. The 18-module stack was connected to 2 identical LED spotlights but going through 2 LED electronic controller/drivers. Twenty hours after inoculation the stacks were able to directly power the bespoke lighting system. The electrical energy produced by the 15-module stack evolved with usage from ≈280 mW (≈2.650 V at ≈105 mA) at the beginning to ≈860 mW (≈2.750 V at ≈300 mA) by the end of the festival. The electrical energy produced by the LED-driven 18-module stack increased from ≈490 mW at the beginning to ≈680 mW toward the end of the festival. During this period, illumination was above the legal standards for outdoor public areas, with the 15-module stack reaching a maximum of ≈89 Lx at 220 cm. These results demonstrate for the first time that the MFC technology can be deployed as a direct energy source in decentralised area (e.g. refugee camps).

## Highlights

• Urine fed microbial fuel cell stacks were used as a direct power source.
• Maintaining individual cells at high potential enables stable series connections.
• A stack continuously and directly powered LED spotlights in the field.
• A stack was connected to the lights through a LED-driver to limit cell reversal.
• Directly connecting LEDs to a stack is more stable than using LED drivers.

## 1 Introduction

The first report on microbial fuel cell (MFC) was published over 100 years ago [1], yet the technology only caught the attention of scientists with the discovery of microbes capable of electron transfer without external redox mediators [2], [3]. For the last 2–3 decades, the technology has made significant progress in terms of power output and details of technology development are comprehensively covered in review papers [4], [5]. MFCs transform chemical energy in organic matter directly into electricity through bacterial metabolism. A vast range of organic matter both in solid and liquid forms can be used as MFC feedstock (fuel), which includes various types of domestic and industrial waste. Its dual functionality, i.e. simultaneous waste treatment and energy generation, makes the technology stand out amongst other renewable energy technologies [6]. Moreover, MFCs can be used for the recovery of resources such as phosphorus [7], nitrogen [8], potassium and copper [9], and as bio-sensors detecting various elements, such as bioactive compounds (e.g. formaldehyde) in drinking water [10] or volatile fatty acids [11], as described in the review from Su [12]. Furthermore the same working principles of the MFC technology can be used, with external power input (microbial electrolysis cell), for producing useful products such as hydrogen [13], [14] or to desalinate water [15], [16].

With technological advancement, there is a realisation that the MFC technology can be used to utilise a rich source of untapped energy i.e. wastewater. Urine has been identified as an ideal fuel for MFCs as it contains a wide range of organics and nutrients [17], [18]. An individual can produce up to 2L of the fuel a day and so in environments where crowds of people are concentrated in one place, a MFC system could be an essential tool for sanitation, treatment and energy generation. Such environments are unpredictable and field trials are imperative to understand the limitations and allow the technology to develop. Glastonbury is one of the world’s most popular music festivals with hundreds of thousands of people camping in a field for 5 days, usually at the end of June. The first MFC Pee Power® trial at the festival was in 2015 where a 330L ceramic-based MFC system lit a male-urinal for up to 10 users at any time and in the meantime processed the urine [19]. With each year the aim has been to improve the efficiency of the system by reducing the MFC footprint whilst lighting larger urinal structures for more users. At Glastonbury 2016, a larger urinal, accommodating up to 18 users at any given time, was powered by a smaller MFC system (reduced in volumetric footprint by a third) [20]. During the 2017 trial, the Pee Power® urinal increased in size to 40 users, a number of challenges were identified, and it was apparent that energy management and feed control needed to be optimised. These were the foci of the current study, where the target for the Glastonbury 2019 field trial was to develop a reliable system that operated stably, providing illumination to European standards for up to 40 people at a time, without energy management circuitry.

When planning for the application, there are two main MFC designs that should be considered; (i) those with exchange membranes and (ii) those without i.e. membrane-less MFCs. The self-stratifying MFCs (S-MFC) are membrane-less and were employed for Glastonbury 2016, 2017 and in the current study. Usually, membrane-less MFCs have an airbreathing cathode that acts as the interface between the electrolyte and the atmosphere. In this configuration, the cathode’s aerobic side catalyses the oxygen reduction reaction (ORR) whereas its anaerobic side sits in direct contact with the electrolyte. A biofilm develops on the anaerobic side of the cathode and acts as a bio-interface limiting the oxygen penetration in the electrolyte, thus, enhancing the activity of the electroactive communities [21]. However, the scalability of this design is limited because the cathode is parallel to the liquid/air interface. Therefore, any size increase of the electrode surface area implies a linear increase of the surface area footprint of the reactor. The S-MFC design alleviates this limitation by having a much higher surface area of electrode for an equivalent footprint. This is due to the fact that the cathode is perpendicular to the electrolyte/air interface. This results in a high surface area of electrode to volume of electrolyte ratio whilst maintaining short diffusion distances between the electroactive communities and the electrolyte [22], [23]. In S-MFCs, cathodes are positioned between 2 and 8 mm above the anodes and are immersed in the electrolyte to about ¾ of their height [24]. S-MFCs exploit a phenomenon observed in any liquid column colonised by life, the chemical and biological stratification of the column under biological activity [22], [25]. The principle is to employ the capacity of microorganisms to maintain two environments with different redox potentials separated by a redoxcline that acts as a sort of “autogenic and transient liquid membrane”. As a result, S-MFCs are scalable in length and width with minimal performance losses [22], but also in height [26], [27].

**Fig. 1:**
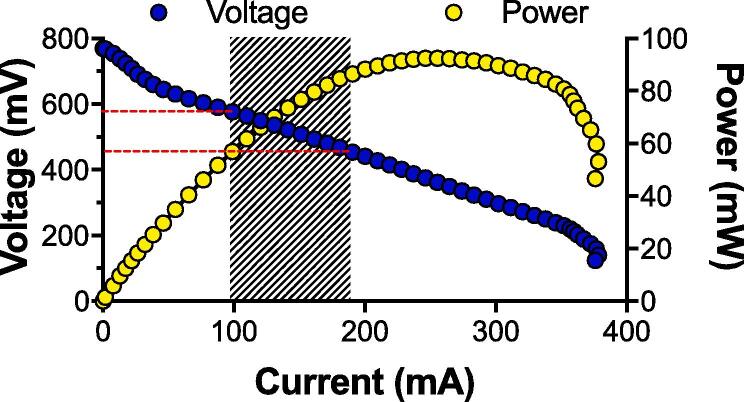
Polarisation curve of a single matured S-MFC module filled with untreated urine. The target for maintaining stability is indicated by the shaded area. (1-column fitting figure).

The present study was driven by the idea of developing the simplest MFC system and employ it as a source of energy in an off-grid context. Following past advances in the domain of self-powered sanitation solutions [19], [20], [28], the objective was to further develop MFC systems towards being battery-free [29]. The main obstacle to overcome is the MFC voltage reversal phenomenon observed when electrically connecting units in series [30], [31]. To avoid this, all successful studies so far have connected units in parallel, or connected stacks in series/parallel combinations [32], [33], and always employed power management systems with energy buffers such as capacitors [23], [34], [35] or batteries [20], [36]. Therefore, the challenge for a battery-free MFC system is to reach sufficiently high voltages, through electrical series connections without any voltage reversal. MFC systems that are implemented to power applications are often operating near the maximum power transfer point (MPT) to maximise energy production. However, under such conditions, the stability and resilience can only be maintained through the use of power management system. The approach developed here was to operate MFCs, electrically connected in series, at a higher potential and a lower current than their respective MPT, thus, maintaining resilience to perturbations encountered in the field (Fig. 1). The hypothesis is that this would enable the assembly of MFC stacks to directly and stably power applications, thus, minimising the cost of the system by avoiding voltage reversal. The strategy to maintain the potential of the modules to a specific range is to limit the current drawn by the application. This implies that either the application has to be limited or the stack as to be scale-up to avoid the application drawing a current close to the MPT of the stack. Since the module’s design could be classified as having a high electroactivity [37], the challenge in developing a stable system was to balance the stack configuration to the maximum energy consumption of the application and deliver the required service.

To verify the hypothesis, the work investigated the use of S-MFCs treating urine to directly power the lighting of a urinal. The strategy was (i) calibrate a lighting system powered directly by a stack of S-MFCs under controlled conditions; (ii) implement the resulting self-lit system for testing in the field (Glastonbury Festival), under uncontrolled operating conditions. The focus was to ensure stability of the stack in a series connection during operation. The aim was to demonstrate how the microbial fuel cell technology could be applied as a direct energy source in decentralised areas. In addition to providing standard lighting and in fitting with the spirit of Glastonbury Festival, the system was also developed to power public engagement activities including neon signage and small computer games.

## 2 Materials and methods

### 2.1 Controlled conditions: Laboratory reactors construction and operation

#### 2.1.1 S-MFC module construction

The S-MFC design employed results from previous studies on urine-fed S-MFCs [22], [26], [38]. A module comprises 41 cathodes placed 5 mm above 31 anodes. Because they were submerged in the same electrolyte, all the electrodes of a module were connected in parallel. The urine level in each module arrived to approximatively ¾ of the cathode height which is the optimum height [36]. Each of the 2 mm thick cathodes had a projected surface area of 126 cm^2^ (42x300mm) and was made by hot pressing (280 °C) a carbonaceous mixture onto a 316 stainless steel mesh (8x8 mesh; MeshDirect, UK). The mixture comprised activated-carbon (AC) and polytetrafluoroethylene (PTFE) with a ratio of 80% AC and 20% PTFE [29]. The loading was of 186 ± 7 mg*_AC/PTFE_*.cm^−2^. Each module had a total cathodic geometric surface area of 5166 cm^2^. Due to the nature of the system (ca. ¾ of the cathode are exposed to the electrolyte on both sides), the total cathodic wet surface area corresponded to 7750 cm^2^. The anode electrode in each module consisted of a carbon veil sheet (1000 mm × 300 mm; 10 g.m^−2^; Technical Fibre Products Ltd, Cumbria, UK) folded down to a geometric area of 150 cm^2^ (300 mm × 50 mm). Each anode incorporated a stapled strip of stainless-steel mesh to operate as current collector. Overall, a single S-MFC module comprised a total of 1 kg of activated carbon, 9.3 m^2^ of carbon veil and a displacement volume of 6.9 ± 0.1 L.

#### 2.1.2 S-MFC cascade assemblies

**Fig. 2:**
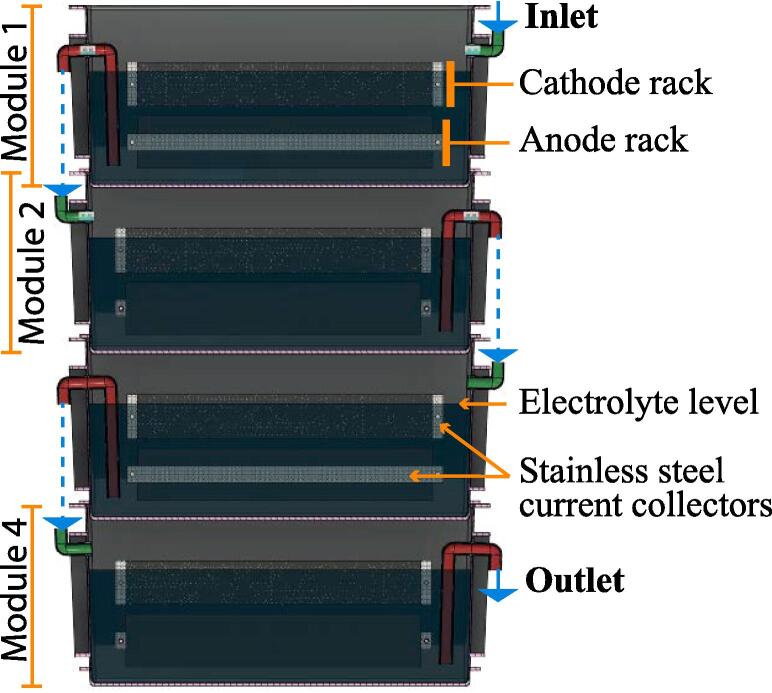
Side view of a cascade comprising 4 S-MFC modules. (1-column fitting figure).

A cascade is defined as a set of modules where the effluent of one module feeds into the next downstream module. At first, the same 4-module cascade (Fig. 2) from a previous study was employed [29]. All the modules of a cascade were electrically connected in series. Following this test, a 5-module cascade and a 6-module cascade were built by adding modules below the initial 4-module cascade and were also connected electrically in series. Based on the previous study [29], the cascades were fed 2.8 ± 0.1 L of urine every 8 h. When more modules were added to the cascade, the feeding regime was shifted to one pulse of 2.8 ± 0.1 L every 6 h. As the cascade was pulse-fed, a module had its volume renewed once every third feed, hence, once every 24 h or once every 18 h. Although this retention time is longer than intended for the design (≈2-8 h HRT per module) [20], there was not enough urine for shorter retention times (this would not be an issue during the field trial). The urine was collected daily from a tank pooling together the urine donated by anonymous individuals. By the time the urine was fed to the cascade, it had gone through partial hydrolysis resulting in increased pH ranging between 8.5 and 9.3.

#### 2.1.3 Experimental setups

The inoculation of the cascade had already been achieved during a previous experiment [29]. The voltages of the cascade and of each of its modules were monitored using an acquisition system (Agilent LXI 34972A; Farnell, UK). The data were logged every 2 min. The current produced by the cascade was measured by monitoring the voltage drop across a wire of a known resistance (0.00281 Ω).

The cascades were connected to purpose-built LED strips comprising from 1 to 8 LEDs (Samsung LM561C, 4000 K) electrically connected in parallel. The LED strips were characterised by linear sweep voltammetry (LSV) under a two-electrode configuration (potentiostat Biologic SP-50) with the reference electrode channel and the counter electrode channel short-circuited.

When the 4-module cascade was shifted towards the 5 and 6-module configurations, all the modules were connected in parallel to avoid cell reversal [30], [32], [39]. At this stage, the cascade was connected to a programmable DC electronic load (BK Precision 8500, B&K Precision Corp., USA) maintaining a constant voltage of 470 mV during 100 h. Once the current output stabilised, the modules of the cascade were electrically connected in series and directly connected to LED strips. The laboratory investigation focused on finding an equilibrium between the number of modules electrically connected in series and the numbers of LEDs that could be directly powered without showing cell reversal or instability. The configurations that were tested are summarised in Table 1.

**Table 1:** Summary of the tested configurations depending on the number of modules electrically connected in series and the number of LED connected in parallel. (1-column fitting table).

	4 modules	5 modules	6 modules
x4 LEDs	C4-L4	C5-L4	----
x8 LEDs	C4-L8	C5-L8	C6-L8

### 2.2 Field trial: Stack configurations and experimental setups

#### 2.2.1 Stacks configuration

**Fig. 3:**
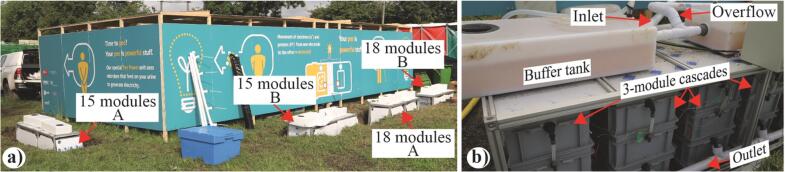
Pictures illustrating the setup deployed during the field trial. Picture (**a**) shows how the four stacks were distributed around the back of the 40-person pissoir, which comprised 2 cubicles of 5 m × 5 m. Picture (**b**) is a close-up of the “15-module B” stack showing the 3-module cascades and the hydraulic connections of the system. (2-column fitting figure).

A previous trial was carried out in 2017 with a urinal of the same capacity (40 people) and size (10 m × 5 m). However, there were a number of lessons learned from that trial that improved the structural design in the current study. In 2017, the system comprised a single, large stack (12 2-module cascades) fed by only 4 of the 12 available troughs. This configuration proved to be suboptimal: (1) collecting the totality of the urine was impossible, (2) the volume of collected urine was insufficient to adequately feed a 24-module stack, and (3) the passive urine distribution between all cascades was not homogeneous and resulted in underperformance (total of ≈ 1.1 W). Based on this experience, the approach adopted in 2019 was to have four S-MFC stacks distributed around the structure, each with its own buffer tank and feeding mechanism. This enabled (1) collecting more urine and having a better buffer capacity, and (2) having a homogenised flow distribution. Two stack configurations were tested, a 15-module and an 18-module configuration. The S-MFC modules employed were the same as the one tested in the laboratory (Fig. 2). Fig. 3 illustrates how the setup was deployed. All four stacks comprised cascades of 3 modules (Fig. 3b) instead of 2 modules like in the 2017 field-test. The two 15-module stacks (15M_S-MFC) comprised 5 cascades of 3 modules, and the two 18-module stacks (18M_S-MFC) comprised 6 cascades of 3 modules. The cost for building one 15-module stack is reported in the Table S1 in the supplementary material. The total cost for such a stack was £2460.17 including the labour for fabricating the modules that accounted for 44% of the total cost. However, since the system was deployed by a group of researchers and technicians without counting the working hours, the cost of the deployment could not be calculated. Moreover, the cost of the structure and the urinal was not taken into consideration due to the partnership with our industrial collaborators. Nevertheless, the costs for fabricating a single module comprises £83.35 for the materials and £71.79 for the student’s labour.

#### 2.2.2 System configuration and operating conditions

Although separated, the four stacks acted as sub-units of the same system. To ensure regular feeding, purpose built 40 mm ID electro-valves were mounted between the buffer tanks and the cascades of each stack. The energy required to actuate the electro-valves was provided by the 15-module A stack (15M_S-MFC_A; Fig. 3a). The 15M_S-MFC_A stack fulfilled three functions: (1) powering the electro valves; (2) continuously powering a purpose-built LED signage encouraging participants to use the pissoir; (3) directly powering a microcomputer (GameBoy Color, Nintendo). To obtain these three functions, the 15M_S-MFC_A stack was electrically connected as two independent stacks. The two first rows of S-MFC modules were connected to a purpose-built power management circuitry hosting a battery (Samsung INR18650-25R; 3.6 V; 2500 mAh), with both modules of a cascade connected in parallel and all five cascades connected in series. Following previous results [29], the last row of the 5 cascades (third modules) were electrically connected in series and directly powering the Gameboy Color. The aim was to offer the public an interactive and engaging platform.

The management circuitry contained a harvester and continuously powered the LED signage whilst timing and powering the feeding of all four stack. To introduce autonomy to the system, custom electro-valves were built for feeding the stacks. The valves were actuated by ‘HS-755MG’ Giant Scale servo motors (HS-755MG, Hitec Inc., Japan) and each had its own microcontroller. The servo motors actuated the 40 mm PVC ball valves that enabled the urine in the buffer tank to flow to the manifold that distributed amongst the cascades of the stack. Power management circuitry sent commands to actuate individual valves in sequence from the first to the last every 60 min. Such sequential feeding, as opposed to feeding all stack at once, prevented too much current being drawn from the battery. Each of the servos had an individual control circuitry which was turned off until receiving a command to actuate the valve. An actuation was completed within 1 s during which the servo draws between 450 mA and 650 mA, therefore requiring 2.25–3.25 W of power. Then the servo’s microcontroller entered deep sleep mode for 29 s during which no power was drawn. Once the microcontroller woke up, the servo was powered to close the valve. After feeding, the electro-valve was powered down for 4 h until the next feed. These 30 s bursts provided 3.35 ± 0.13 L of urine per cascade for the 15M_S-MFC stacks, and 3.13 ± 0.18 L per cascade for the 18M_S-MFC stacks. This feeding pattern resulted in a hydraulic retention time (HRT) of roughly 24 h per stack/cascade and a treatment capacity of roughly 104 L and 124 L for the 15M_S-MFC stacks and the 18M_S-MFC stacks, respectively.

The field experiment monitored and compared two stack configurations, the 15M_S-MFC_B stack and one of the two 18-module stacks (18M_S-MFC_B; Fig. 3a). Two approaches were investigated, first the LED spotlights were directly connected to the stack (15M_S-MFC_B), and secondly LED-Drivers were acting as buffers between the stack and the LED spotlights (18M_S-MFC_B).

The 18M_S-MFC stacks were assembled with 6 cascades because the chosen LED-driver had an optimal running voltage of 1.5 V. Hence, since (1) the objective was to directly and continuously power LED spotlight without the use of a power management system, and (2) the running voltage of a S-MFC directly powering an application was roughly 500 mV [29]; each of the 18M_S-MFC stacks were electrically configured as two stacks of 3 cascades, each electrically connected in series, with all 3 modules within a cascade being electrically connected in parallel. Each of these 3-cascade sub-stacks was connected to a single LED-driver (MCP1643; Microchip Technology Inc.) powering a single LED spotlight. The LED-drivers had the option of limiting the current delivered to the LED to 25 mA, 75 mA or 125 mA.

#### 2.2.3 Data capture

An acquisition system (Agilent LXI 34972A; Farnell, UK) monitored the voltage of each cascade and each stack by logging data every 4 min. The current produced by the cascade was measured by monitoring the voltage drop across a known resistance (0. 1 Ω) positioned in the junction boxes connecting the stacks to the LED spotlights.

Urine samples were collected in duplicate and analysed in triplicate. The samples were filtered upon collection (0.2 µm pore size membranes) and immediately placed in a freezer. Samples were taken during the automated feeding every 24 h to match the hydraulic retention time of the systems. The inlet samples were taken in the buffer tanks. The outlet samples were taken at the end of the common outlet pipe, 15 s after the cascade started releasing the treated waste. The chemical oxygen demand (COD) analyses were performed using the potassium dichromate oxidation method (COD HR test vials, Camlab, UK) with 0.2 mL of inlet and outlet. The vials were heated at 150 °C during two hours and cooled to room temperature before the measurements were taken using an MD 200 photometer (Lovibond, UK). The results are presented as average values, comprising duplicates of each sampling time together with the triplicates of analysis.

## 3 Results

### 3.1 Calibration of LED lighting and stack configuration

**Fig. 4:**
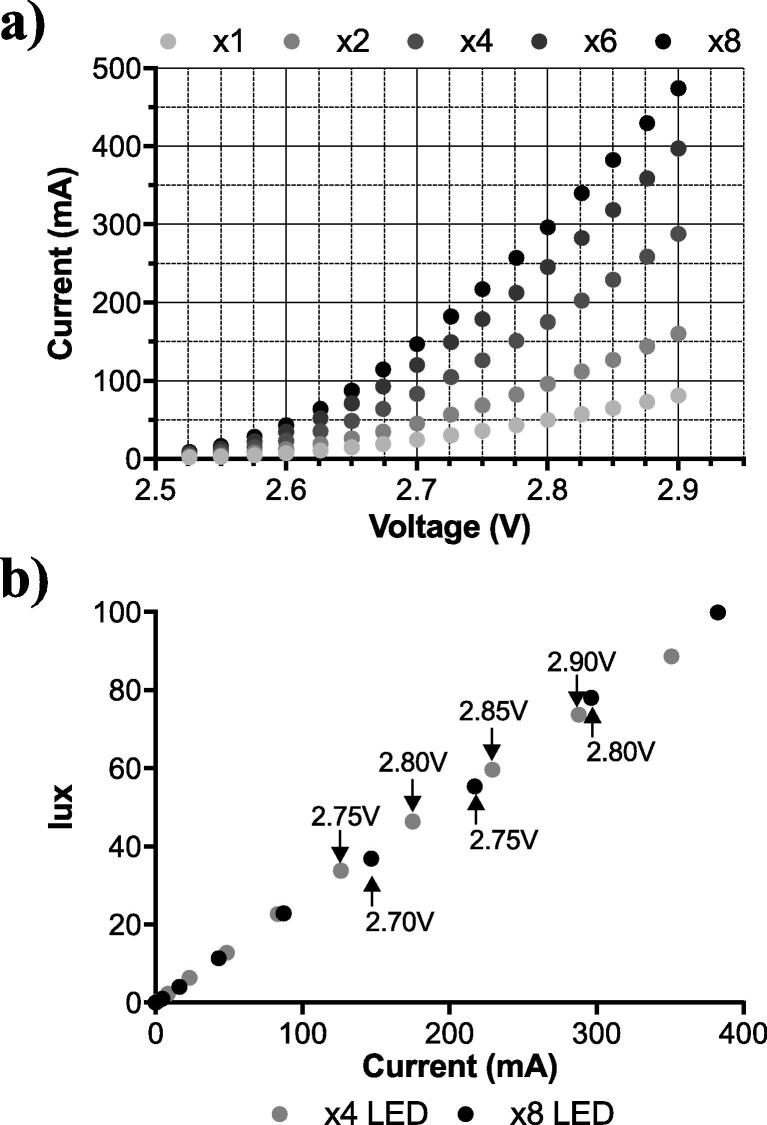
Results of the operating conditions and performance of different numbers of LEDs (LM561C). Figure (**a**) exponential current consumption depending on the provided voltage. Figure (**b**) illumination of the LEDs depending on the current. (1-column fitting figure).

The calibration experiment aimed at finding an equilibrium between the number of LEDs and the electrical configuration of an S-MFC stack whilst maintaining stable and continuous operation. LEDs connected in parallel were characterised, firstly by running a linear sweep voltammetry experiment (LSV) to measure the current consumption under a given voltage, and secondly by measuring the illumination in a black box at 92 cm (0.01 V increments; 2.55 V to 2.90 V). The voltage sweep was stopped at 2.9 V, voltage at which the tested LED efficiency decreased due to thermal losses. The results show the non-linear and positive relationship between the voltage provided and the current consumed by the LED (Fig. 4a). Although the relation between the number of LED and the current consumption at a given voltage should be linear, results show a slight deviation between 6 LEDs and 8 LEDs. This could be due to an increased resistance with the increased soldering. Conversely, the relation between the illumination measured at 92 cm and the current consumed by the LED is linear and independent from the number of LED employed (Fig. 4b). Hence, the same illumination will be obtained at a given current, which implies providing a higher voltage for lower number of LED (Fig. 4b).

Assuming that the stable operating voltage of a single S-MFC module ranged between 450 mV and 600 mV (Fig. 1), the corresponding current produced would be between 95 mA and 190 mA. This operating range is for a single matured module under optimal conditions (i.e. full of fresh urine). A previous study showed that the same module under running conditions would produce between 87 mA and 163 mA at the same voltage range [29]. It is important to note that 450 mV is near the maximum power transfer point (388 mV; MPT). Such a module would have a small resilience to the perturbations occurring in the field. Based on these polarisation results, the estimation was that a stack comprising modules electrically connected in series should be able to sustainably output a current of 95–120 mA. With this current level, a LED system would produce roughly 20–25 Lx (Fig. 4b). This implies that depending on the number of LEDs, the stack should be running at a voltage of either 2.70 V or 2.65 V if connected to x4 LEDs or x8 LEDs, respectively. In order to reach 2.70 V, a stack should comprise 6 modules electrically connected in series and operating at around 450 mV each. Following the same logic, a stack aiming at providing 2.65 V should comprise 5 modules electrically connected in series and operating at around 525 mV each (average voltage between 450 mV and 600 mV).

The aim was to evaluate the stability of a S-MFC stack against the uncontrolled conditions found in the field. Following the LED characterisation results, two spotlight configurations were investigated (4 LED and 8 LEDs). Initially, the 4-module cascade previously used for another experiment was tested [29]. Afterwards, a module was added at the bottom of the cascade to create the 5-module cascade. This module was also tested against a 4-LED and 8-LED spotlight. Finally, a 6-module cascade was tested with an 8-LED spotlight. The assumption was that if the output of a cascade electrically connected in series was stable during the laboratory investigation; then a stack deployed in the field, with uniformity between cascades connected in series, should also be stable.

**Fig. 5:**
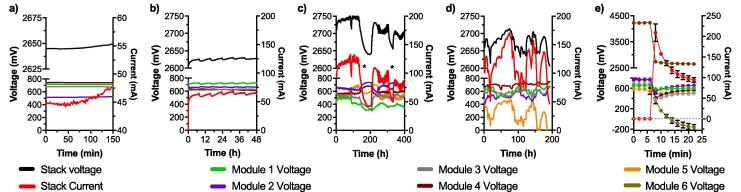
Stack electrical performance under the five tested conditions (see Table 1). (**a**) a cascade of 4-modules in series powering a 4-LED spotlight (C4-L4); (**b**) a cascade of 4-modules in series powering a 8-LED spotlight (C4-L8); (**c**) a cascade of 5-modules in series powering a 4-LED spotlight (C5-L4), the * indicate starvation periods during which the cascade was not fuelled; (**d**) a cascade of 5-modules in series powering a 8-LED spotlight (C5-L8), (**e**) a cascade of 6-modules in series powering a 8-LED spotlight (C6-L8). The error bars in (**e**) stand for the value range of the 2 successive experiments performed with the same cascade. (2-column fitting figure).

The results show that the electrical output of the 4-modules stack was stable with either the 4-LED or the 8-LED spotlights (Fig. 5a,b). Under a feeding regime of 1 pulse of 2.8 L every 8 h, the stack generated 48 mA at 2.65 V and 63 mA at 2.62 V when connected to 4 LEDs (C4-L4) or to 8 LEDs (C4-L8), respectively (Table 2). According to the LED characterisation results (Fig. 5b), this corresponded to 13 Lx and 18 Lx (at 92 cm) for the C4-L4 and C4-L8 conditions, respectively. Although minor, the 25 mV decrease in the voltage output between C4-L4 and C4-L8 conditions resulted in a 30% current output increase for the latter condition. However, in both cases the voltages of the individual modules were between 600 mV and 700 mV and could be considered as open circuit conditions. These results illustrate that 4 S-MFCs modules electrically connected in series could not provide a voltage high enough to match the requirements of the LEDs. In such configuration, more LED could have been added in order to push the stack voltage lower and extract more current from it. However, due to the operating voltage range of the employed LED, such a logic would only work up to a stack voltage of 2.60 V at which the 8 LEDs would not switch ON. However, such changes would not have been significant in terms of illumination.

**Table 2:** Summary of the relation between stack voltage under load and the current consumed by the LED (data from the LSV experiment) depending on the tested configurations (see Table 1). (1-column fitting table).

Tested conditions	Stack voltage (V)	LED current consumption (mA)	Light intensity (Lx)*
C4-L4	2.646 ± 0.002	46.1 ± 1.2	13.2
C4-L8	2.624 ± 0.009	62.2 ± 7.0	17.5
C5-L4	2.722 ± 0.019	102 ± 16.6	26.1
C5-L8	2.705 ± 0.006	156.3 ± 9.6	40.5
C6-L8	2.737 ± 0.011	199.54 ± 14.5	51.1

* Measured at 92 cm.

Based on these results and on the LED characteristics, it was assumed that decreasing the voltage of the individual modules to provide higher current could only be achieved by adding a module in the series electrical connection. Adding a module would result in an increased stack voltage, thus, in a higher current consumption from the LED spotlights, thus, in a lower voltage and higher current production from the individual modules. This assumption was first tested with a cascade of 5-modules directly connected to a 4-LED spotlight (C5-L4). During the initial 114 h of the run, the stack stably produced 118 ± 7 mA at a voltage of 2.740 ± 0.008 V (Fig. 5c). Since the system, as a whole (c.a. the S-MFC stack and the LED spotlight), is meant to face uncontrolled conditions, the resilience of the cascade was tested through a 72 h period of starvation (first * in Fig. 5c). After this period, a pulse feed of 10 L was provided prior shifting the system to its previous feeding pattern (1 pulse every 6 h). Over the following 100 h, the performance was slightly lower with a stack voltage of 2.716 ± 0.010 V and a current of 96 ± 9 mA. However, the system output was stable over that period, demonstrating the resilience of the stack to starvation. A second starvation test was performed for a period of 30 h without feeding (second * in Fig. 5c). Conversely to the first starvation test, no initial burst feed was given to restart the system that was directly shifted toward its initial feeding regime. The system recovered from its starvation state and had a stable output during the following 110 h run with a voltage of 2.702 ± 0.010 V and a current of 86 ± 8 mA. The results of this experimental run demonstrate that, overall, a stack of 5 S-MFC modules electrically connected in series can (1) continuously and reliably power a 4-LED spotlight at a relatively high voltage (Table 2) without cell reversal (Fig. 5c), and (2) recover from starvation, demonstrating resilience.

Building on these results, the cascade of 5 modules was connected to an 8-LED spotlight (C5-L8) under the same feeding regime of one pulse of 2.8 L every 6 h. Beside the initial voltage drop due to a lack of feeding (t = 10 h to t = 22 h; Fig. 5d), the voltage and current slowly increased for the first 82 h reaching 2.704 V and 168 mA, respectively). However, after reaching this maximum, the outputs of the stack rapidly decreased to 2.645 V and 85 mA after about 50 h (Fig. 5d). The voltage measurements of the modules indicated that the drop was due to the last module of the stack (module 5, positive port of the stack) that ended up with a voltage of 118 mV. Although not characterised as cell voltage reversal, the behaviour of this last module clearly indicated that the performance of the stack was unstable, as illustrated by the voltage of module 2 that showed an initial voltage decrease at t = 107. This suggests that module 5 was providing most of the current, at a low voltage, whilst the other modules were providing less current at a higher voltage (Fig. 5d). Whilst displaying a low voltage the other modules had an average voltage output of 645 ± 47 mV. An attempt to recover the initial performance was performed by doubling the volume fed to the stack at t = 123 h. This resulted in the voltage increase of module 5 and in the voltage decrease of the other modules (Fig. 5d), with the stack voltage and current outputs reaching 2.704 V and 158 mA. However, these outputs were not stable over time and the voltage of module 5 started decreasing again; At t = 162 h, module 5 went into cell voltage reversal reaching −24 mV. A second recovery attempt was performed at t = 164 h by doubling the feeding volume of two consecutive feed-pulses. Although this resulted in a partial performance recovery, the system displayed a new cell reversal at t = 185 h. This experimental run illustrated that the load, consuming more than roughly 150 mA at 2.7 V, was too high to maintain the stability across all modules of the stack (Table 2). Indeed, drawing a higher level of current from the stack lead one of the modules (module 5 in Fig. 5d) entering cell reversal due to a shift beyond its MPT. It can be assumed that this increase of the drawn current deceased the voltage of the module resulting in a voltage increase of the other modules to compensate the overall stack voltage, which in turn increased the current drawn from a single module (positive feedback-loop). Following these results, another module was added to the cascade to power the same 8-LED spotlight (C6-L8). The main result is that the system was not stable for more than 4 min and after 8 min the last module of the cascade entered cell voltage reversal (Fig. 5e). As the stack was able to deliver a higher voltage, the LED consumed more current than an individual module could provide, resulting in cell reversal. This illustrated that providing higher voltage by having more units connected in series is not the optimal approach when powering an application that that can draw proportionally more current with higher voltages.

### 3.2 S-MFC stack configurations to directly power LED in the field

The challenge in assembling a stack as the direct energy source for lighting applications is to adjust the number of modules electrically connected in series. The higher the number of modules, the higher the voltage, however, the higher the provided voltage, the higher the current consumed by the LED. A higher current consumption could cause the drop of the voltage of some modules in the stack. This would result in some modules being at a high voltage and providing a low current, whilst others being at a low voltage and providing a high current. An unbalance that will increase with time and subsequentially lead to cell reversal or at least to an unstable system unable to self-recover (Fig. 5d,e). Conversely, if the number of modules connected in series is low, so will be the voltage of the stack. Such a low voltage resulting in a low current production, hence, in a stable but underperforming and inefficient system where all modules would display near open circuit conditions (Fig. 5a,b).

Results have shown that the C5-L4 condition displayed a balance, between the number of LEDs and the number of modules, that enabled stability (Fig. 5c). However, the illumination was insufficient to light one of the two 20-person compartments of the urinal (Table 2). Since the system was deployed in an outdoor environment, the aim was to generate around 30 Lx at floor level, between the 20–50 Lx range of the European standard for outdoor lighting (EN 12464-2, 2007 [40]). This implied increasing the number of LED, which in turn meant increasing the number of modules electrically connected in parallel. Based on the LED characteristic (Fig. 4b) of 30 Lx and on the limited height available between the troughs outlets and the soakaway, the aim was for a system of 300 mA. This meant that the stack was assembled with 5 cascades electrically connected in series, each cascade comprising 3 modules electrically connected in parallel (Fig. 3b, 15M_S-MFC_B). Based on the results (Fig. 5, Table 2) we were extrapolating that the 15M_S-MFC_B would produce roughly 2.700 V and 300 mA to power two 6-LED spotlights, hence producing around 20–50 Lx over the 5 m × 5 m 20-person cubicle. As demonstrated by the laboratory investigation, if the voltage of a stack increases slightly, the current drawn by the LED could lead the modules to enter a cell reversal state. Hence, the possibility to limit the current drawn by LED through the use of LED drivers was explored. As explained in Section 2.2.2, the 1.5 V operating voltage of the LED drivers lead to the development of 6-cascade stacks (Fig. 3a, 18M_S-MFC) electrically subdivided into 2 sub-stacks of 3 cascades. Each of these sub-stacks were connected to a single 6-LED spotlight. These resulted in the second 20-person cubicle comprising four 6-LED spotlights. During this trial, only the stacks 15M_S-MFC_B and 18M_S-MFC_B had their electrical performance monitored.

### 3.3 Field comparison of two system configurations

The core focus of the field trial, besides deploying a green solution to sanitation in decentralised area, was to investigate the potential of having lights continuously and directly powered by a MFC stack. Two solutions were explored: directly connect a stack to two 6-LED spotlights (stack 15M_S-MFC_B, Fig. 3a); connecting two 9-modules sub-stacks to one 6-LED spotlight with a LED driver to limit drawn current and avoid cell reversal (stack 18M_S-MFC_B, Fig. 3a). Both stacks were powering an identical pair of 6-LED spotlights to which they were connected 22 h after inoculation, during daytime (≈13:00 h). Although inoculated 22 h prior to being connected to the spotlights (see §1.1 in Supplementary Material), regular feeding commenced 44 h h after inoculation (i.e. enough fuel was harvested). This was because the urinal was next to the main stage (a.k.a. Pyramid Stage) that opened on the second day of the Festival. Nevertheless, the electro-valves were regularly actuated regardless of the availability of fuel (see §1.2 in Supplementary Material).

#### 3.3.1 Direct connection to spotlights

**Fig. 6:**
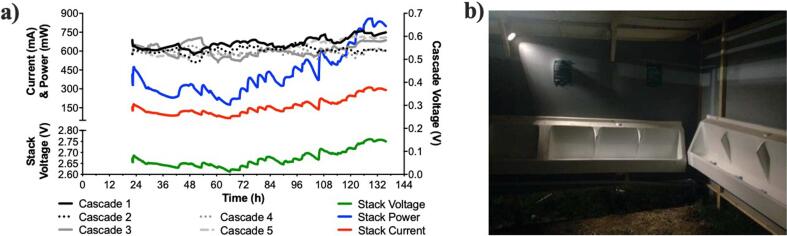
Electrical outputs of the 15M_S-MFC_B stack (**a**) that was directly powering two 6-LED spotlights during the 5 days of the Glastonbury Music Festival 2019. The right picture (**b**) illustrates the illumination obtained by one of these 6-LED spotlights that was lighting half of one of the two pissoir compartments. (2-column fitting figure).

The 15-module stack was designed to be directly connected to the lighting system without any electronic support, thus, demonstrating the sustainability of the technology. During its initial running time the stack 15M_S-MFC_B was outputting roughly 100 mA at 2.640 V (Fig. 6a). As showed in Fig. 6, the outputs were relatively stable until regular feeding occurred. Since the stack voltage was low, so was the current drawn by the LED.

Once fed on a regular basis (t = 44 h), the electrical output of the 15M_S-MFC_B stack decreased down to roughly 80 mA at 2.620 V during the following 22 h. Then, at t = 66, both the voltage and the current continued increasing until the end of the trial at t = 136 h (Fig. 6a) where the stack was outputting 301 ± 9 mA at 2.756 ± 0.003 V (average between t = 126 h and t = 136 h). These measured values indicate that during the last 10 h of the trial the 15M_S-MFC_B stack was outputting 829 ± 19 mW with a maximum of 859 mW at t = 128 h (311 mA at 2.760 V; Fig. 6a). During this period, the voltages of individual cascades were oscillating between 510 mV and 610 mV. These results show that (1) the stack was continuously maturing over the trial period of 136 h, and (2) the S-MFC maturation and operation were not impaired by the LED. Although the maximum temperature increased from 21 °C at the time of the inoculation (Fig. S3, Day 1 at 14 *h*00) to 30 °C on day 5 (Fig. S3; corresponding to t = 120 in Fig. 6), the power output of the 15M_S-MFC_B stack was stable on day 6, when the maximum temperature during the day was of 22 °C. Therefore, it is difficult to evaluate the impact of temperature on the performance of the maturing system. It should be noted that the lowest temperatures during operation were around 13–14 °C. However, it can be assumed that that these relatively high daytime temperatures were beneficial for the system maturation.

#### 3.3.2 Impact of LED-drivers

**Fig. 7:**
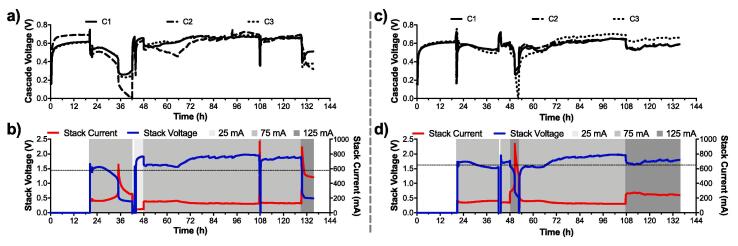
Electrical outputs of the two 9-modules sub-stack of the 18M_S-MFC_B stack. (**a**) and (**b**) are the temporal data of the sub-stack 1 (LD_1), and (**c**) and (**d**) are the temporal data of the sub-stack 2 (LD_2). (**a**) and (**c**) are the voltages output of each of the three cascades within a sub-stack, whilst (**b**) and (**d**) are the performance of the sub-stack themselves. The grey areas in (**b**) and (**d**) indicates under which current limitation the LED drivers were set. In (**b**) and (**d**) the dashed lines indicate the optimal operating voltage below which the LED drivers were not working properly. (2-column fitting figure).

Conversely to the 15-module stack, the 18-module stack employed a LED-driver to limit potential cell reversal. Initially, one of the sub-stacks displayed a stable output (LD_2; Fig. 7c,d), the other sub-stack (LD_1) was unstable (Fig. 7b) and went into cell reversal (Fig. 7a). During this phase, both LD_1 and LD_2 had the LED driver limiting the current to 75 mA at roughly 1.5 V. The cell reversal of LD_1 was triggered by the decrease in the sub-stack voltage due to the limited fuel renewal at this early stage. As the voltage lowered, the LED driver drew more current (t = 31 h in Fig. 7b) to maintain the constant 75 mA output to the LEDs. However, the current output increased, from roughly 150 mA to 230 mA) resulting in cascade 2 going into a cell reversal (Fig. 7a). On the other hand, LD_2 had a constant current output of 153 ± 9 mA at 1.624 ± 0.083 V between t = 22 h and t = 42 h (Fig. 7d). At this time an unsuccessful attempt to shift the LED-driver of LD_2 to 125 mA was made prior to shifting back to 75 mA output. In the meantime, the LED driver of LD_1 was shifted to 25 mA output to recover from the cell reversal state. Once the stacks were under regular feeding, a second attempt to shift the LED-driver of LD_2 to 125 mA was performed at t = 48 h. However, the cascade 3 of LD_2 went into cell reversal. As for the initial phase of LD_1, the LED driver compensated the low voltage by drawing more current from the stack, which drove LD_2 to pass beyond the maximum power transfer point of its power curve (Fig. 1). These results indicate that although the LED driver limits the current drawn by the LED, when the S-MFCs are unmatured, it acts conversely to LEDs by drawing more current when voltage input decreases, which in turn push the S-MFC toward cell reversal.

After setting the output of LC_1 to 25 mA for 5 h (t = 43 h; Fig. 7a,b), the cascade voltages indicated that the cascade 2 had recovered from the cell reversal. At t = 50 h, the LED driver of LD_1 was shifted to 75 mA output. Initially, cascade 2 voltage decreased until t = 65 when it started recovering and finally displayed a voltage value similar to the other two cascades. The average voltage of the three cascades, during the last 50 h under the 75 mA condition, was of 662 ± 31 mV (Fig. 7a) with the stack series connection averaging at 1.896 ± 0.047 V and 129 ± 4 mA (Fig. 7b). The average voltage of LD_2 cascades, during the last 30 h under the 75 mA condition, was 651 ± 26 mV (Fig. 7c) with the stack series connection averaging 1.933 ± 0.036 V and 125 ± 3 mA (Fig. 7d). Although the electrical outputs were similar between the two stacks under the 75 mA condition, they diverged when the LED-driver of both LD_1 and LD_2 were shifted to 125 mA conditions. The voltage and current of LD_1 stack, measured at the input of the LED-driver, did not make much sense since the voltage of the stack was lower than the voltage of the individual cascades that were electrically connected in series. This implies that when the voltage provided to the LED-driver is lower than the 1.5 V threshold, the driver operation impedes the reading. Interestingly, the calculated power between the 75 mA and 125 mA conditions were similar (247 ± 1 mW and 245 ± 5 mW, respectively). Nevertheless, the LD_1 cascade voltages indicate that cascade 2 was decreasing linearly, hence, entered a process of cell reversal. Conversely, LD_2 displayed stable outputs with an average current of 253 ± 10 mA and an average voltage of 1.737 ± 0.049 mV, over the last 28 h of the trial under the 125 mA condition (Fig. 7d). The cascade voltages were also stable with cascade 1 and 2 averaging 567 ± 18 mV and cascade 3 averaging 645 ± 184 mV.

#### 3.3.3 Light intensity

The fact that the 15M_S-MFC_B stack was outputting roughly 300 mA during the last 20 h implied that each LED was consuming around 25 mA (two 6-LED spotlights). In comparison, the LED-driver powered by the LD-2 stack delivered 125 mA to a single 6-LED spotlight, hence resulting in 21 mA per LED. The LED-driver powered by the LD-1 stack was also providing 21 mA per LED after t = 128 h. However, conversely to LD_2 that had a stable electrical performance, the cascades in LD_1 were showing signs of a future cell reversal, thus, indicating that the system was unstable under these conditions. During most of the trial, the LED-drivers of both LD_1 and LD_2 were limiting the current to 75 mA, implying that during this period each LED of a 6-LED spotlight was provided 12.5 mA. Although not measured in the field, the illumination obtained from one of the 6-LED spotlight can be seen in Fig. 6b. The fact that the LEDs were encased into a reflector increased illumination by focussing the light where it was needed. Earlier measurements made in the laboratory showed that adding a reflector increased the illumination at 2 m from 12 Lx to 26 Lx. Further testing after the field trial showed that two 6-LED spotlights powered at 310 mA produced an illumination of 40–45 Lx each at 2.2 m with a maximum of 89 Lx where the light beams were overlapping (Table 3.). Although below the standard of 100 Lx found in building, the light level produced during the trial were well into the remits of 20–50 Lx recommended for outdoor public areas (EN 12464–2, 2007, [40]), thus, following the European legislation on the matter and achieving a good level of comfort for the users.

**Table 3:** Summary of the illumination measured in a black room, at given current,s by the 6-LED spotlights. Measures made with a domed sensor (Sunche HS1010). (1-column fitting table).

Tested conditions	Provided current (mA)	Illumination (Lux)
1 spotlight	25	4
	75	18
	125	33
2 spotlights	310	89 44/45*

* Per spotlight.

#### 3.3.4 Treatment efficiency

**Fig. 8:**
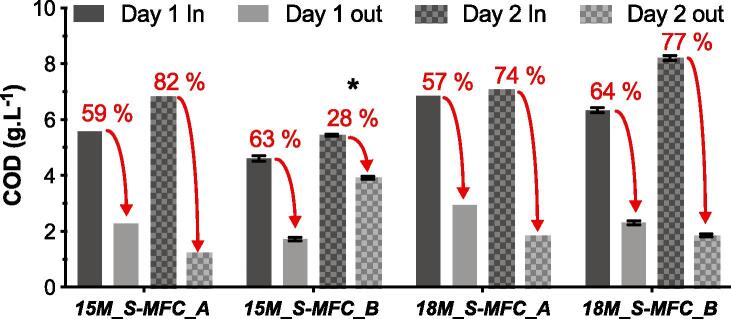
COD concentration measured in the buffer tanks (In) and in the outlet (Out) of all four stacks (Fig. 3a). These measures were performed during 2 consecutives days at a HRT of 24 h, except for *. (1-column fitting figure).

All four stacks were sampled for COD concentration over two day to evaluate the treatment efficiency at the 24 h hydraulic retention time of the cascades (from t = 70 h to t = 118 h). Samples from the stacks 15M_S-MFC_A and 18M_S-MFC_A were only analysed once (no error bars). The first observation is that the COD concentration of the urine in the buffer tanks varied independently from the sampling time or the buffer tank (Fig. 8); the overall average COD concentration of the inputs was of 6.37 ± 1.12 g.L^-1^. This result illustrates the great variability the collected urine has in its chemistry over the course of 2 days. During this sampling period the feeding of stack got interrupted between t = 98 h and t = 106 h, as shown by the voltage drop (Fig. 6a). After identifying the cause of the drop, the feeding was shifted to 1 feed every 2 h instead of once every 4 h with the aim of enabling the stack to recover from the decline. However, if the 15M_S-MFC_B stack recovered its performance, this feeding impacted the HRT of the stack by flushing away the volume of urine that was sampled at the inlet 24 h earlier. Moreover, this implied that the COD was measured on a volume of urine that had a 14 h residence time. Due to this and to the chemical variability of urine, the result of 28% COD concentration decrease is indicative (Fig. 8*). Nevertheless, the four systems had decreased the COD concentration by 61 ± 3% on day 1 and 77 ± 4% on day 2, illustrating that at the end of the trial the stacks were still maturing. The treatment efficiencies were relatively constant for each stack, on a day to day basis, illustrating the constancy of the treatment of the MFC technology at a given HRT (24 h). However, the COD content of the urine had a high variability between days and buffer tanks. This variability of the urine COD content is due to numerous factors such as diet, hydration, life style or the environment of the users [17]. Therefore, as the treatment efficiency was relatively constant, the COD concentration of the effluent wasn’t (2.03 ± 0.54 g.L^-1^). The COD results of this study are in line with the removal rates published by a previous study [20], which has measured a 75% COD decrease with a HRT of 22 h, under laboratory conditions.

## 4 Conclusions

A previous study has demonstrated that an off-the-shelf application otherwise utilising batteries could be continuously and directly powered by an MFC stack. However, this was done under controlled laboratory conditions. The present study developed the approach further towards application (1) by investigating how to calibrate the application (LEDs) to the stack, and vice versa, in order to develop a stable system, and (2) developing a prototype system, as a sanitation solution for decentralised areas, and test it during a field trial. The laboratory investigation confirmed that in order to avoid cell reversal in uncontrolled environments, the units that are electrically connected in series should have their voltage maintained relatively high so that their electrical output is below their maximum power transfer point (MPT). With regard to the chosen application, since the current consumption of LED is “exponentially” increasing with increasing voltages, the number of units electrically connected in series should be limited to prevent individual voltage levels to drop too low, thus, having the unit electrical output going beyond MPT. Besides, having too high of load (i.e. number of LED) also leads to MFC voltage reversal of one of the units electrically connected in series. Overall, the operating conditions of the LEDs drive the number of S-MFC module to be connected in series, and the number of modules connected in parallel allow to tailor a specific illuminance output. The results from the field trial have shown that when these parameters are met, the LEDs act as a power management system that progressively increased the load on the stack. Hence, results have demonstrated that directly connecting the LED to the S-MFC stacks enables the system to effectively self-adjust to the uncontrolled environment in which it was deployed. Conversely, employing a LED-driver to limit the current drawn is not optimal for the MFC maturing period, as it requires frequent adjustments for the LED-driver to be efficient, although, on a matured system, an LED-driver could be employed to provide constant illumination levels. However, a harvesting system comprising a battery was used during the trial to actuate the electro-valves. Hence, passive feeding is an aspect that should be investigated further for the S-MFC system to be electronic-free and fully autonomous.

## CRediT authorship contribution statement

**Xavier Alexis Walter:** Conceptualization, Methodology, Data curation, Investigation, Writing - original draft, Writing - review & editing, Supervision. **Jiseon You:** Writing - review & editing, Data curation. **Jonathan Winfield:** Investigation, Writing - review & editing. **Ugnius Bajarunas:** Investigation, Data curation, Writing - review & editing. **John Greenman:** Conceptualization, Supervision, Writing - review & editing. **Ioannis A. Ieropoulos:** Conceptualization, Supervision, Funding acquisition, Writing - review & editing.

## Declaration of Competing Interest

The authors declare that they have no known competing financial interests or personal relationships that could have appeared to influence the work reported in this paper.
