# Supplementary material for: From the lab to the field: Self-stratifying microbial fuel cells stacks directly powering lights
Source: Appl Energy. 2020 Nov 1;277:115514. doi: 10.1016/j.apenergy.2020.115514 (PMC7567022; doi:10.1016/j.apenergy.2020.115514)
Supplement: Supplementary data 1 [file mmc1.docx]

**From the lab to the field: self-stratifying microbial fuel cells stacks directly powering lights.**

Xavier Alexis Walter^1^*, Jiseon You^1^, Jonathan Winfield^1^, Ugnius Bajarunas^1^, John Greenman^1^ and Ioannis A. Ieropoulos^1^*

^1^ Bristol BioEnergy Centre, Bristol Robotics Laboratory, T-Block, Frenchay Campus, University of the West of England (UWE), Bristol, BS16 1QY, United Kingdom.

*Corresponding authors: Xavier Alexis Walter and Ioannis A. Ieropoulos

Bristol BioEnergy Centre (BBiC), Bristol Robotics Laboratory, T-Block, Frenchay Campus, University of the West of England, Bristol, BS16 1QY, United Kingdom

Tel: +44 (0)117 3286788

E-mail: [xavier.walter@brl.ac.uk](mailto:xavier.walter@brl.ac.uk) / [ioannis.ieropoulos@brl.ac.uk](mailto:ioannis.ieropoulos@brl.ac.uk)

**Email address of the authors:**

Dr X. Alexis Walter: [xavier.walter@brl.ac.uk](mailto:xavier.walter@brl.ac.uk)

Dr Jiseon You: [Jiseon.You@uwe.ac.uk](mailto:Jiseon.You@uwe.ac.uk)

Dr Jonathan Winfield: [Jonathan.Winfield@uwe.ac.uk](mailto:Jonathan.Winfield@uwe.ac.uk)

Mr. Ugnius Bajarunas: [Ugnius.Bajarunas@uwe.ac.uk](mailto:Ugnius.Bajarunas@uwe.ac.uk)

Prof. John Greenman: [john.greenman@uwe.ac.uk](mailto: john.greenman@uwe.ac.uk)

Prof. Ioannis A. Ieropoulos: [ioannis.ieropoulos@brl.ac.uk](mailto:ioannis.ieropoulos@brl.ac.uk)

**1 Inoculation of the S-MFC stack for the field trial**

Inoculating 4 large stacks with a total of 66 modules representing a total volume of 455 L could not be done by using the effluent of existing S-MFCs. In addition, transporting onsite such a large volume of activated sludge was challenging. The solution pursue was to employ the leachate/supernatant of the mobile pit-latrine toilets already deployed onsite. However, it took time to accumulate the needed volume which was achieved roughly 20 h prior the pissoir had to be opened to the public. After keeping the stack under open circuit condition for 1h and reaching roughly 650mV, all the unit within each stack were electrically connected in parallel. The 15M_S-MFC stacks were placed under a load of 2.25 Ω and were outputting around 288 mA at an average 648 mV. The 18M_S-MFC were placed under a load of 1.75 Ω and were outputting around 358 mA at an average 628 mV. Figure S1 illustrates the inoculation behaviour of the 15M_S-MFC stack that was then directly connected to the two 6-LED spotlights. At t=5 h after inoculation the stack 15M_S-MFC_B a 2.25 Ω load was added in parallel to the previous one, resulting in an overall 1.125 Ω load. Under this condition, the 15M_S-MFC_B stack was producing 512 mA (Fig. S1). This result illustrates that the load could have been higher from the start. Compared to a previous field trial with S-MFCs [1], the inoculation with pit-latrine leachate worked well. The difference was that for this 2019 trial, the pit-latrine leachate had not been previously settled in a tank as for the 2016 trial. This year, the leachate contained a lot of suspended solid and corresponded to concentrated blackwaters since it has not been diluted by flushing water but by urine.

***Figure S1:*** *Electrical behaviour of the stack 15M_S-MFC_B after inoculation with pit-latrine leachates.*

**2 Electrical outputs of the stack used for the demonstration**

Although the setup deployed comprised four hydraulically and electrically independent stacks (Fig. 3a), it acted as one system. This was due to the stack 15M_S-MFC_A that was powering and controlling the actuation of the electro-valves of all four stacks. This was achieved by the power management circuitry harvesting the energy produced by the 10-module sub-stack of stack 15M_S-MFC_B (see §2.2.2). The initial experimental monitoring of the feeding process showed that once the stack was fed regularly (t=20h after inoculation; Fig. S2) it could stably power the LED signage and at t=27h after inoculation also one electro-valve once every hour (Fig. S2). At t=49h the automated feeding was switched off to evaluate the time needed to reach full charge. Results indicate that the full charge of the battery took around 20h (Fig. S2).

***Figure S2:*** *Voltage measurement of the battery within the harvester, and current output of the 10-modules sub-stack of the 15M_S-MFC_A stack. The dark grey area indicates when the stack was fed regularly (once every 4 hour).*

**3 Temperature variation during the field trial**

The maximum temperature increased from 21°C on the day of inoculation to 30°C on the fifth day of the trial (Fig. S3). Conversely, the minimum temperature was stable during the all duration of the trial (Fig. S3). The data were taken from the archive of the Accuweater.com website.

***Figure S3:*** *Temperature variations during the field trial. The inoculation occurred on Day 1.*

**4 Costs of fabrication**

***Table S1:*** *Overall costs for the build of a 15-modules stack*

|  | **Parts** | **Price for 15M_S-MFC (£)** | **Percentage of the total** | |  |
| --- | --- | --- | --- | --- | --- |
| **Material for the modules** | **Cathode** | 267.21 | 10.86 | 50.82 | |
|  | **Anode** | 595.56 | 24.21 |  |  |
|  | **Embodiment** | 387.41 | 15.75 |  |  |
| **Plumbing for the stack** | **buffer tanks** | 80.76 | 3.28 | 5.66 | |
|  | **valve** | 12.92 | 0.53 |  |  |
|  | **PVC pipe and fittings** | 45.48 | 1.85 |  |  |
| **Labour** | **modules (75h)** | 1070.83 | 43.53 | 43.53 | |
|  | **Total (£)** | 2460.17 | 100 | 100 | |

* The cost of labour was calculated using the time taken by one student to fabricate 15 modules whilst being paid at the hourly rate for student temporary work.

References:

[1] Walter XA, Merino-Jiménez I, Greenman J, Ieropoulos I. PEE POWER® urinal II – Urinal scale-up with microbial fuel cell scale-down for improved lighting. Journal of Power Sources. 2018;392:150-8.
